# EpiSNPdb: A Comprehensive Database of Genetic Epistasis Across Multiple Cancer Types

**DOI:** 10.3390/cimb48080753

**Published:** 2026-07-24

**Authors:** Xiaohong Wu, Jianye Yang, Wen Cao, Jiaxin He, Congcong Min, Xiaohui Niu, Yuan Quan, Jing Gong

**Affiliations:** 1 Hubei Key Laboratory of Agricultural Bioinformatics, College of Informatics, Huazhong Agricultural University, Wuhan 430070, China; wu.xiaohong@webmail.hzau.edu.cn (X.W.); jyyang@webmail.hzau.edu.cn (J.Y.); caowen20@mails.ucas.ac.cn (W.C.); he_jiaxin@webmail.hzau.edu.cn (J.H.); mincongcong@webmail.hzau.edu.cn (C.M.); niuxiaoh@mail.hzau.edu.cn (X.N.); 2 College of Biomedicine and Health, Huazhong Agricultural University, Wuhan 430070, China

**Keywords:** epistasis, single nucleotide polymorphism, pan-cancer, database

## Abstract

Increasing evidence shows that epistasis, defined as interactive effects between genetic loci, may contribute to the missing heritability of cancer. However, systematic genome-wide epistasis identification in cancer remains challenging. Here, by leveraging genotype and clinical data from 380,983 samples in the UK Biobank, we identified 202,032 candidate epistatic single nucleotide polymorphism (epiSNP) pairs associated with cancer risk across 16 cancer types. Notably, multivariable Cox regression identified 123 epiSNP pairs with significant interaction effects on overall survival, suggesting that interaction-level genetic signals can provide prognostic information beyond individual SNP effects. Through functional analysis of the 202,032 candidate epiSNP pairs, we identified 7152 pairs supported by gene co-expression data and 12,326 pairs with protein–protein interaction (PPI) evidence. By mapping epiSNP pairs to corresponding gene pairs and then linking these gene pairs to drug–target databases, we identified 1040 epistatic gene pairs with FDA-approved drug–target records. Additionally, through KM survival analysis of the candidate epiSNP pairs, we detected 7068 pairs significantly associated with patient overall survival. Finally, we constructed an open-access database, EpiSNPdb, to facilitate cancer epistasis research.

## 1. Introduction

Genome-wide association study (GWAS) has advanced our understanding of the genetic architecture of complex diseases, especially cancer [1]. Despite this advancement, missing heritability in cancer remains insufficiently explained by GWAS-identified risk loci [2]. The limitation of conventional GWAS lies in the fact that although GWAS can capture additive effects of individual genetic loci, it cannot capture the complex interplay between genomic loci influencing disease risk [3].

Epistasis, namely SNP–SNP interaction, is defined as interactive effects between genetic loci and may help to explain missing heritability in cancer [4,5,6,7,8]. Increasing evidence from cancer research indicates that epistasis plays a significant functional role in cancer. For example, our previous work has shown that interaction between rs11777210 in the KBTBD11 gene and rs6983267 in the oncogene MYC increases colorectal cancer risk by 2.83 times [9]. Subsequent genome-wide epistasis analyses in lung cancer further identified significant epistatic SNP pairs. Moreover, incorporating these interactions significantly enhanced the predictive power of the polygenic risk score for patient stratification [10,11].

However, genome-wide epistasis identification is confronted with the following challenges. The exhaustive test of all pairwise SNP interactions across the genome imposes a large computational burden [8,12]. The modest effect sizes of epistatic interactions require sufficient sample size to ensure detection capability [4]. Moreover, existing methods often inadequately control false positives, especially in unbalanced case–control studies [13,14].

Notably, recent advances in biobank-scale genomics and statistical methodologies enable us to overcome the aforementioned limitations. The UK Biobank (UKBB) represents an unprecedented, large-scale genomic resource that combines genotype, lifestyle, and clinical data from over 500,000 participants, enabling powerful integrated analyses [12]. The recently developed SAIGE algorithm can effectively control false-positive rates in unbalanced case–control studies and is well suited for biobank data analysis [15,16]. Moreover, Zhang et al. demonstrated that the epistatic interactions identified by SAIGE can improve polygenic risk prediction in patients [11].

In this study, we systematically identified epistasis across 16 cancer types using genotype and clinical data from 380,983 samples in the UK Biobank. To maximize the retention of potential epistatic signals while managing computational complexity, we first screened candidate epistatic single-nucleotide polymorphism (epiSNP) pairs from GWAS-significant variants (*p* < 0.05) and then applied sensitivity analyses to support robust associations. This approach yielded 202,032 candidate epistatic SNP pairs, of which 71,649 pairs passed sensitivity testing and were designated as SAIGE-significant interactions. Furthermore, based on the 202,032 candidate epiSNP pairs, we performed functional annotation, protein–protein interaction (PPI) mapping, gene co-expression analysis, drug–target association, and survival analysis to explore the biological and clinical relevance of these interactions. Finally, we constructed EpiSNPdb (https://gonglab.hzau.edu.cn/EpiSNPdb/#/, accessed on 21 July 2026), a user-friendly database to help further investigate genetic interactions in cancer and generate hypotheses for risk assessment and pharmacologic exploration.

## 2. Materials and Methods

### 2.1. Data Collection

We obtained imputed genotype data, health-related records, and clinical information of approximately 500,000 participants from UKBB (https://www.ukbiobank.ac.uk/, accessed on 1 January 2025) [12]. Protein–protein interaction (PPI) data were obtained from the STRING database (https://cn.string-db.org/, accessed on 13 June 2025) [17]. The data on drugs and their targets were collected from DGIdb v5.0 (https://dgidb.org, accessed on 20 October 2025) [18], TTD (https://ttd.idrblab.cn/, accessed on 9 October 2025) [19], and DrugBank v6.0 (https://go.drugbank.com, accessed on 10 October 2025) [20]. The information on combinatorial drugs and their indicators was retrieved from DrugCombDB [21]. This study is based entirely on germline genetic variants from the UK Biobank and does not include somatic mutations.

### 2.2. Data Processing

To minimize the impact of population stratification on epistasis analysis, we selected the largest subgroup, White British individuals from UKBB as study objects [22]. Based on cancer diagnosis codes (ICD-9 and ICD-10) and cancer histology codes, we extracted and screened cancer samples from UKBB. Participants with a recorded ICD-9 or ICD-10 cancer diagnosis were classified as cases, whereas participants without any cancer diagnosis were defined as controls. To ensure sufficient statistical data, we selected 16 cancer types with >1000 case samples for epistasis analysis [14,23].

We performed quality control on samples and SNPs through strict filtering criteria using PLINK [24]. We excluded aneuploid samples [12], the samples with heterozygosity rates > 3 SD from the mean [25,26], those with an overall missing call rate of >5% [27], and those with inconsistent recorded and biological sex [28]. Additionally, we also excluded the SNPs with a missing rate of >5% [29], those with minor allele frequency (MAF) ≤ 0.001 [12], and those with Hardy–Weinberg equilibrium *p*-values < 1 × 10^−6^ [30].

### 2.3. Identification of Genome-Wide Risk Loci Associated with Cancer Susceptibility

To identify genome-wide risk loci associated with cancer susceptibility, this study used a case–control design, with cancer patient samples as cases and non-cancer samples as controls. Patient age, gender, and the top 40 principal components were used as covariates to account for potential confounding factors [31]. SNPs associated with cancer susceptibility were identified with a *p*-value < 0.05 as a threshold via GWAS [10,32,33,34]. The GWAS was performed using fastGWA-GLMM in the GCTA software (Version v1.11) [35].

### 2.4. Identification of epiSNP Pairs

Considering the computational complexity of testing all possible interaction combinations, this study investigated only pairwise interactions. To identify potential epiSNP pairs, we performed an epistasis analysis on candidate SNPs identified by the GWAS using ‘--epistasis’, ‘--fast-epistasis boost’ and ‘--fast-epistasis joint-effects’ functions in PLINK [36,37]. For the epistasis test, we used --epistasis with --make-pheno covariate.txt 2, where the covariate file included age, sex, and the top 40 principal components; the --make-pheno parameter adjusts the phenotype for covariates prior to the epistasis scan. For the boost regression test, we used --fast-epistasis boost with --epi1 1, which adjusts the *p*-value threshold for inclusion of interaction pairs in the main report. For the joint-effects test, we used --fast-epistasis joint-effect with --je-cellmin 3, which ensures that each cell in the 3 × 3 × 2 contingency table contains at least three samples, thereby reducing unstable estimates from sparse cells. Ultimately, we identified 202,032 epiSNP pairs with *p*-value < 1 × 10^−5^ by PLINK for subsequent analysis [38]. This threshold was chosen to maximize sensitivity and capture potentially important signals, providing a comprehensive resource for further discovery. To provide a higher-confidence subset of interaction candidates, we applied Benjamini–Hochberg (BH) multiple-testing correction to the results of each PLINK method separately. Pairs with FDR < 0.05 were designated as “high-confidence findings,” while those passing only the initial threshold (*p* < 1 × 10^−5^) were designated as “exploratory candidate interactions.”

### 2.5. Sensitivity Analysis

To improve the robustness of statistical estimation under the case–control unbalanced condition, we first excluded epiSNP pairs where any cell in the 3 × 3 × 2 contingency table contained fewer than three samples [39]. We then applied SAIGE as a sensitivity filter for the remaining pairs. SAIGE uses Firth-corrected logistic regression with saddlepoint approximation to control for case–control imbalance and sample relatedness, making it well suited to handling the sparse contingency tables that frequently arise when samples are stratified by joint genotypes—a scenario analogous to rare-variant analysis where this approach has been validated and recommended [15,40,41]. Pairs with *p* < 0.05 in SAIGE were considered as passing the sensitivity test [11]. Throughout this manuscript, we refer to the 202,032 pairs from initial PLINK screening as “candidate epiSNP pairs” and the 71,649 pairs that remained significant after SAIGE sensitivity analyses as “SAIGE-significant interactions”.

### 2.6. Calculation of OR

We calculated epiSNP pair OR values via Fisher’s exact test [9]. For each pair, we report the OR of the double-homozygous genotype combination (ORaabb) relative to the reference genotype [42]. Pairs with a nominal *p* < 0.05 in Fisher’s exact test were further examined [43]. Among these, pairs with ORaabb > 1 were described as showing a risk-increasing pattern, and those with ORaabb < 1 were described as showing a risk-decreasing pattern [44]. The results were visualized using three-dimensional bar graphs.

### 2.7. Genomic and Functional Annotation of epiSNP Pairs

We performed genomic annotation via ANNOVAR software (Version 2019Oct24) [45]. To further delineate the gene pairs containing epiSNP pairs, we mapped gene pairs to a PPI network from the STRING database [17] and matched these gene pairs with drug information from DGIdb [18], TTD [19], DrugBank [20], and DrugCombDB [21]. In the STRING database, confidence scores were categorized into low (150–399), medium (400–699), and high (≥700) [17].

### 2.8. Gene Expression Correlation Analysis

To investigate the gene expression correlation between two epiSNPs within an epiSNP pair, we performed a Spearman correlation analysis of the expressions of epiSNP pair-containing gene pairs from the TCGA database [46]. |R| > 0.3 and *p*-value < 0.05 were considered as significant correlation [47]. Expression correlations were visualized using scatter plots.

### 2.9. Identification of Survival-Related SNPs and epiSNP Pairs

We analyzed the association between survival time and SNP or epiSNP pair genotypes using additive, dominant, and recessive models in the log-rank test. We encoded SNP genotypes as 0, 1, and 2 in terms of the number of effect alleles [48]. In the additive model, based on the three genotypes (0,1, and 2) of each SNP, patients were divided into three groups; based on the nine genotypes (0_0, 0_1, 0_2, 1_0, 1_1, 1_2, 2_0, 2_1, and 2_2) of each epiSNP pair, patients were divided into 9 groups. Then the differences in survival time among the groups were examined. Similarly, in the dominant and recessive models, based on the number of genotypes of each SNP or epiSNP pair, patients were divided into the same number of groups, followed by the examination of the differences in survival time among the groups.

FDR correction was applied to the log-rank test results, and epiSNP pairs with FDR < 0.05 were selected for Cox regression analysis. Pairs for which the median survival time could not be estimated in any genotype group were excluded.

Multivariable Cox proportional hazards models were fitted with the two SNP main effects, their interaction term, and available covariates. Age and smoking status were included for all cancer types, and sex was additionally included for cancers affecting both males and females. Hazard ratios, 95% confidence intervals, and *p*-values were reported. The proportional hazards assumption was evaluated using Schoenfeld residuals.

### 2.10. Database Construction of EpiSNPdb

All the results obtained in this study were submitted to the MongoDB database (version 3.4.2). The EpiSNPdb (https://gonglab.hzau.edu.cn/EpiSNPdb/#/, accessed on 21 July 2026) was constructed based on the Python Flask (version 1.0.3) framework, and all the data in this database can be browsed, retrieved, and downloaded. EpiSNPdb is run on the Apache2 web server (version 2.4.18). EpiSNPdb has been tested on various web browsers.

## 3. Results

### 3.1. The Overview of Analytical Framework

**Figure 1:**
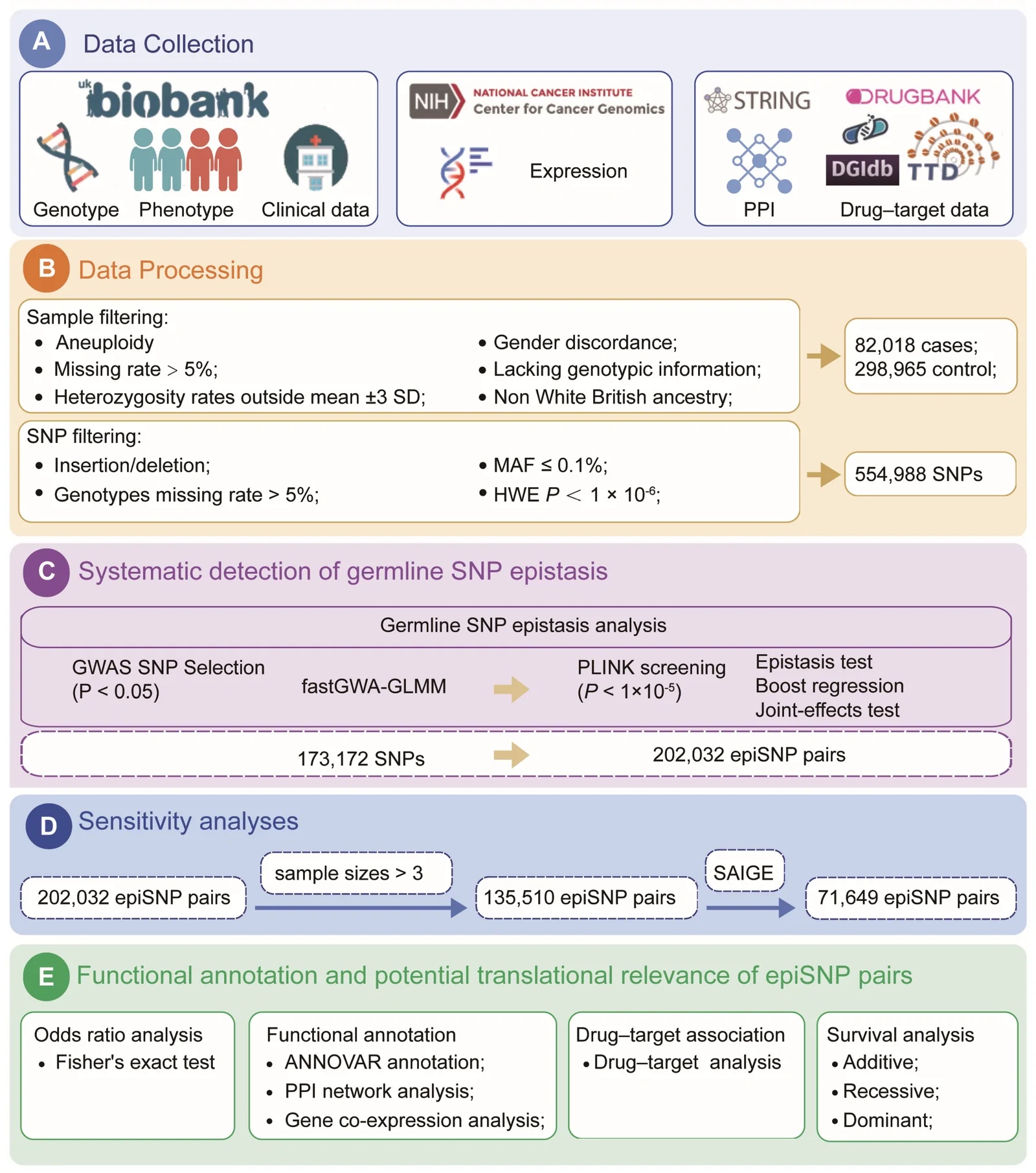
Diagram illustrates the data analysis and database construction in this study. (**A**) Data collection from public resources. (**B**) Quality control of samples and SNPs. (**C**) Systematic detection of germline SNP epistasis. (**D**) Sensitivity analyses. (**E**) Functional annotation and potential translational relevance of epiSNP pairs.

To systematically identify epistasis in cancer, we first obtained imputed genotypes and clinical information from UKBB. To minimize population stratification bias, we only considered the White British ancestry, which is the largest genetic subgroup in UKBB. After quality control, we obtained 554,988 high-quality SNPs from 380,983 samples, including 82,018 cancer patients and 298,965 non-cancer participants (Figure 1). To ensure statistical power for epistasis analysis, we selected 16 cancer types with sample sizes of >1000, with cancer sample sizes ranging from 1122 for liver cancer to 19,207 for breast cancer (Table 1). Our multi-omics analytical workflow comprised three major components: (i) systematic detection of germline SNP epistasis, (ii) sensitivity analysis of epiSNP pairs, and (iii) functional annotation and clinical assessment of candidate epiSNP pairs.

### 3.2. Systematic Germline SNP Epistasis Detection from UKBB Population

**Figure 2:**
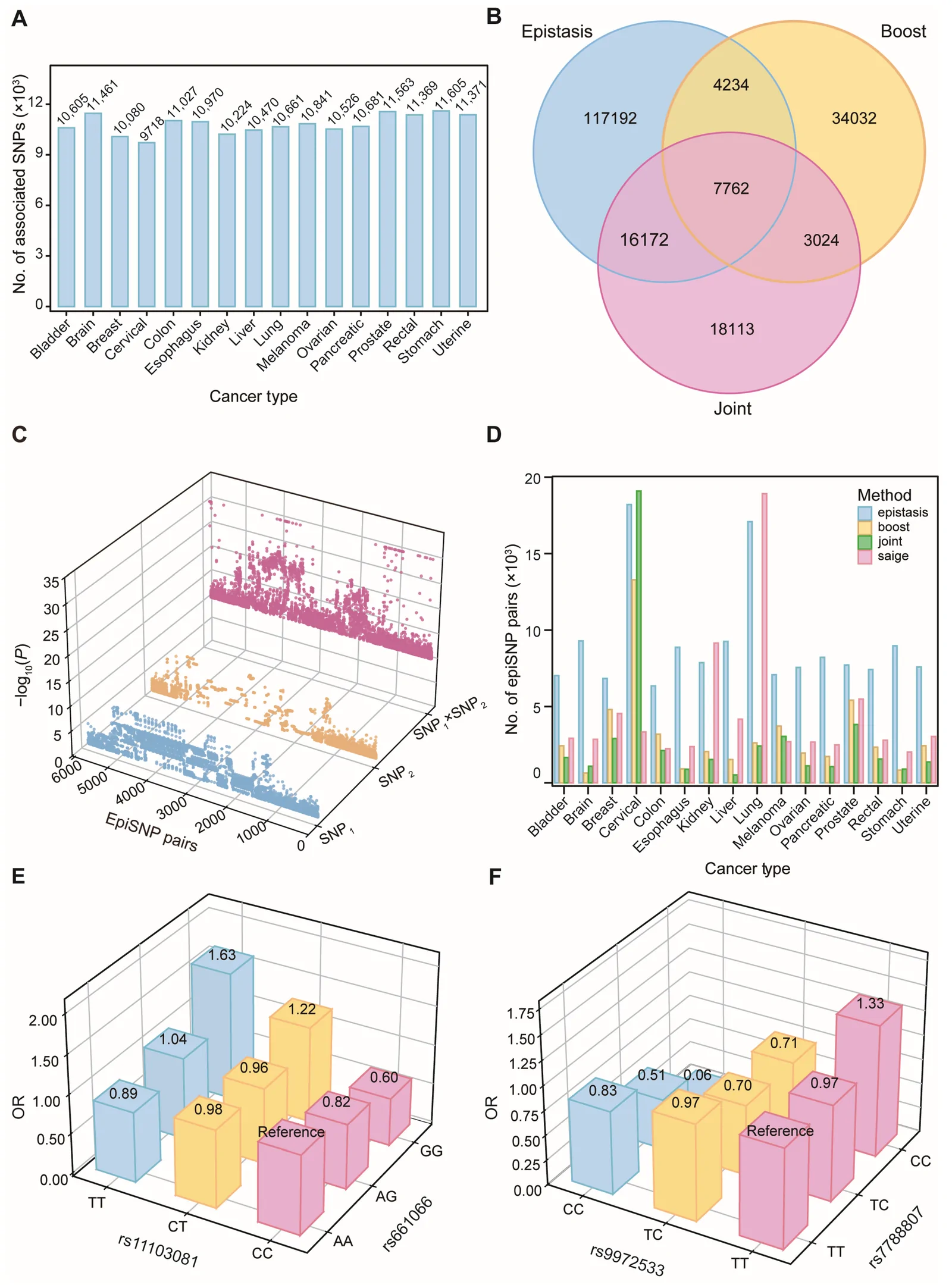
The identification of epiSNP pairs across 16 cancer types. (**A**) Bar chart displays the number of candidate SNPs (*p* < 0.05) identified by GWAS for each cancer type. (**B**) Venn diagram illustrates the overlap of epiSNP pairs detected by three PLINK methods, including epistasis test, boost regression, and joint-effect test. (**C**) A three-dimensional bar chart presents the epiSNP pairs that are statistically significant (*p* < 5 × 10^−8^) at the interaction level but whose individual SNPs do not reach genome-wide significance in GWAS (*p* > 5 × 10^−8^). The X-axis contains three quantitative dimensions, including the single-variant GWAS *p*-value of SNP1, the single-variant GWAS *p*-value of SNP2, and the interaction *p*-value of the SNP1 × SNP2 pair. The Y-axis shows the number of qualified interaction pairs, and the Z-axis shows the -log_10_ (*p*-values) of the individual SNPs or the epiSNP pair, with higher values indicating stronger statistical significance. (**D**) The number of epiSNP pairs identified by three PLINK methods (candidate epiSNP pairs; n = 202,032) and those that passed SAIGE sensitivity analyses (SAIGE-significant interactions; n = 71,649) across 16 cancer types. (**E**,**F**) Representative epistatic patterns from the SAIGE-significant interactions. (**E**) A pattern where the double-homozygous genotype shows an OR > 1 indicates a risk-increasing joint effect. (**F**) A pattern where the double-homozygous genotype shows an OR < 1 indicates a risk-decreasing joint effect. The terms “risk-increasing” and “risk-decreasing” describe the joint genotype rather than individual alleles. OR values for individual alleles are shown alongside the joint OR.

To mitigate the computational burden of pairwise SNP epistasis tests, we first performed a GWAS for each cancer type and obtained 173,172 candidate SNPs (*p* < 0.05) for epistasis analyses, with an average of 10,824 SNPs per cancer type (Figure 2A). Subsequently, we applied three complementary methods (epistasis test, boost regression, and joint-effects test) in PLINK software (version 1.9) to detect epiSNP pairs from 173,172 candidate SNPs. From these candidate SNPs, we identified a total of 202,032 candidate statistical epiSNP pairs using three complementary PLINK-based approaches. Specifically, the standard epistasis test detected 145,360 pairs with significant deviation from multiplicative effects (*p* < 1 × 10^−5^); the boost regression identified 49,052 pairs exhibiting strong interactive effects despite weak individual ones; and the joint-effect test yielded 45,071 pairs where both main and interaction effects were statistically significant (Figure 2B). After Benjamini–Hochberg correction (FDR < 0.05), 28,817 epiSNP pairs remained significant across all methods (6222 from the epistasis test, 6408 from BOOST, and 16,187 from the joint-effects test). These interactions constitute a higher-confidence subset within EpiSNPdb, while the full set of 202,032 pairs was retained as exploratory candidate interactions. Of the 202,032 candidate pairs, 7762 pairs were identified by all three methods. Notably, among all candidate epiSNP pairs, 5929 pairs were statistically significant at the epiSNP pair level, but the individual SNPs comprising them did not reach significance in conventional GWAS (*p* < 1 × 10^−8^) (Figure 2C). This finding underscores the potential value of statistical epistasis analysis in cancer genetics, since it can identify epiSNPs that may be associated with cancer susceptibility through interactive effects.

### 3.3. Sensitivity Analyses of epiSNPs

To control the false-positive rate under the case–control unbalanced condition, we excluded epiSNP pairs where any cell in the 3 × 3 × 2 contingency table contained fewer than three samples and obtained 135,510 candidate epiSNP pairs for subsequent analysis. We then carried out a sensitivity analysis of these pairs using SAIGE, which implements Firth’s bias correction and saddlepoint approximation to maintain statistical robustness. Eventually, we obtained 71,649 nominal-significant epiSNP pairs (*p*-value < 0.05), with an average of 4493 epiSNP pairs per cancer type (Figure 2D). Of the 71,649 nominal-significant epiSNP pairs, 71,411 pairs were specific to a single cancer type, and 238 pairs were shared by two cancer types. Specifically, 18 pairs were shared by brain and esophagus cancers, 219 pairs by lung and cervical cancers, and one pair by esophagus and stomach cancers.

### 3.4. Functional Annotation and Potential Translational Relevance of epiSNP Pairs

To assess the functional prioritization of epistasis on cancer, we performed Fisher’s exact test across eight genotype combinations relative to the reference genotype using the 71,649 SAIGE-significant interactions. We examined the OR patterns of the 71,649 SAIGE-significant interactions by evaluating the double-homozygous OR (ORaabb) relative to the reference genotype. Among these, 36,533 pairs reached nominal significance (*p* < 0.05) in Fisher’s exact test. Of these, 14,181 pairs showed an ORaabb > 1 (Figure 2E), 22,106 pairs showed an ORaabb < 1 (Figure 2F), and 245 pairs showed an ORaabb equal to 1.

**Figure 3:**
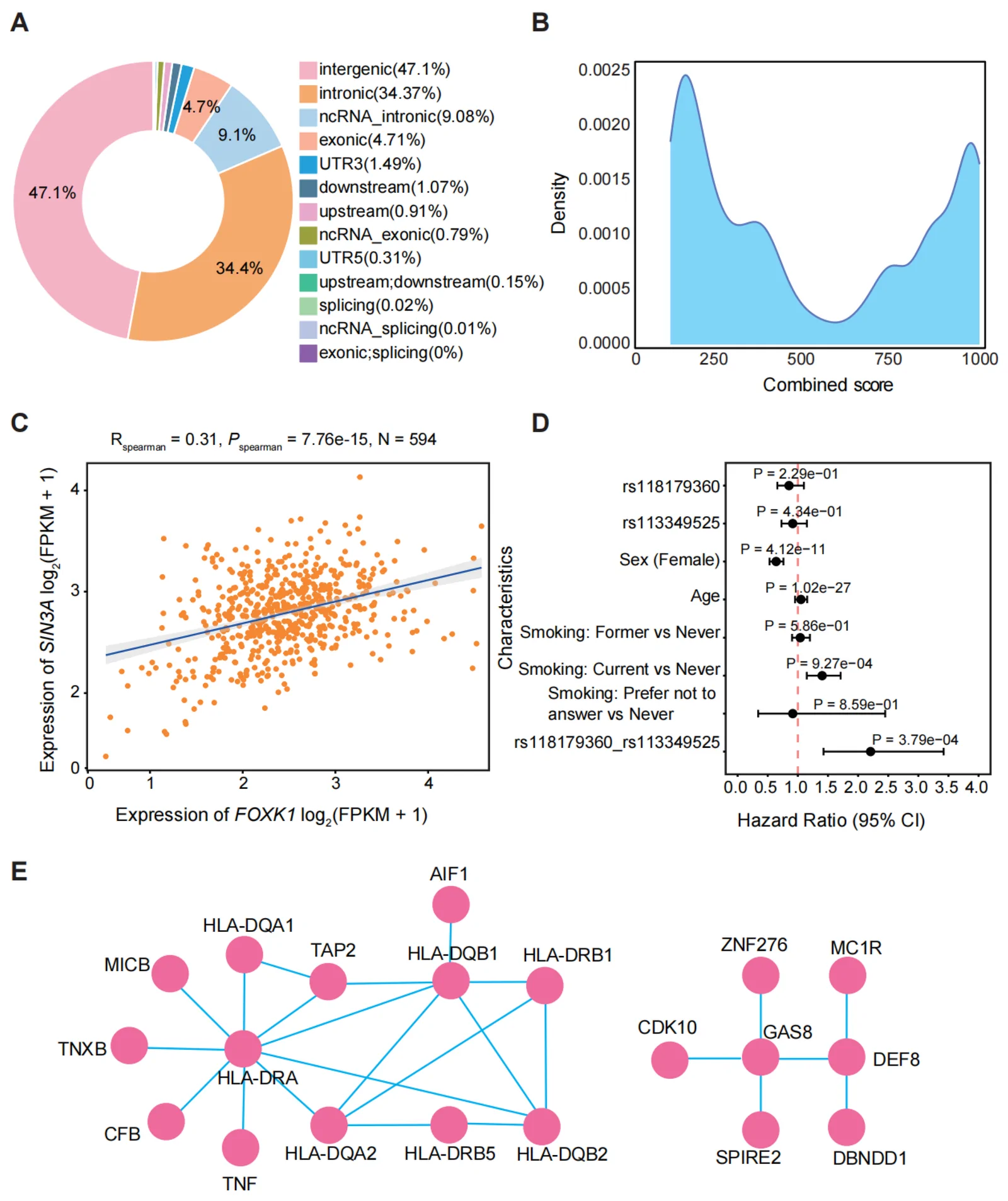
Multi-level functional annotation of epiSNP pairs. (**A**) Genomic distribution of epiSNPs from the 202,032 candidate epiSNP pairs. (**B**) Density plot of PPI confidence scores (150-999) from STRING for epistatic gene pairs. (**C**) Scatter plot of expression correlation between *FOXK1* and *SIN3A* genes based on TCGA RNA-seq data (R_spearman_ = 0.31, *p*-value = 7.76 × 10^−15^, n = 594). The solid line represents the linear regression fit for the expression correlation, and the shaded area indicates the 95% confidence interval of the regression estimate. (**D**) Multivariable Cox regression analysis of prognostic factors for overall survival in liver cancer. The dashed line indicates the hazard ratio (HR) threshold of HR = 1, which serves as the critical reference for evaluating increased or decreased cancer risk. (**E**) Genetic interaction network supported by epistatic, co-expression, and PPI evidence. Only hub genes with degree ≥ 3 and their directly connected neighbors are shown.

To examine the genomic distribution of the 202,032 candidate epiSNP pairs, epiSNPs were annotated to genes using ANNOVAR. The results showed that epiSNPs are located in diverse genomic regions, with the intergenic region containing the largest proportion of epiSNPs (46.50%), followed by the intronic region (33.67%), ncRNA_intronic region (8.80%), and exonic region (6.11%) (Figure 3A).

To explore the potential functional relevance of epistasis, we investigated the co-expression patterns of gene pairs mapped from the 202,032 candidate epiSNP pairs using TCGA RNA-seq data. As a result, 7152 gene pairs showed significant expression correlation (|R| > 0.3 and *p* < 0.05), of which 6305 gene pairs were positively correlated, and 847 gene pairs were negatively correlated.

We further integrated PPI annotation for gene pairs mapped from the 202,032 candidate epiSNP pairs and found that 12,326 pairs were supported by PPI evidence from the STRING database. Among them, 7069 pairs showed medium confidence scores (400–699), 2866 pairs achieved high confidence scores (≥700), and the remaining 2391 pairs showed low confidence scores (150–399), suggesting that our identified epistatic interactions are consistent with known protein–protein interaction networks (Figure 3B). Notably, 1048 epiSNP pairs satisfied three criteria, including nominal-significant epistasis, expression correlations, and documented PPI. For example, we identified a protective epiSNP pair rs7788807_rs9972533 that was associated with reduced lung cancer risk (*P*_joint-effects_ = 2.80 × 10^−6^, *P*_saige_ = 1.49 × 10^−2^, OR_aabb_ = 0.06) (Figure 2F). The corresponding genes, *FOXK1* and *SIN3A*, showed positive correlation in expression (R_spearman_ = 0.31, *p* = 7.76 × 10^−15^) (Figure 3C), and their encoded proteins were linked by a high-confidence PPI (confidence score = 946). These findings provide supportive evidence for potential functional relatedness. Previous studies have reported that the FOXK1–SIN3A complex can act as a transcriptional repressor involved in epigenetic regulation, including genes implicated in tumor progression [49,50,51]. In this study, the observed epistatic interaction between variants mapped to *FOXK1* and *SIN3A* is consistent with a potential regulatory relationship.

To further prioritize interactions with consistent support across multiple complementary evidence sources, we applied a stringent filtering strategy requiring FDR < 0.05, co-expression evidence (*p* < 0.05, |R| ≥ 0.3), documented protein–protein interactions in STRING, and literature-reported cancer relevance. This filtering yielded five interaction pairs (Table 2). Notably, four of these five pairs involve HLA family genes in cervical cancer, with co-expression correlations ranging from 0.65 to 0.83 and STRING confidence scores from 879 to 994, consistent with the established role of antigen presentation in cervical cancer pathogenesis. The SETD9–MIER3 pair in breast cancer, supported by a STRING score of 617 and four independent publications, represents a candidate for further functional investigation.

To evaluate the potential therapeutic relevance of the PPI-supported epistatic gene pairs identified above, we matched the 12,326 gene pairs to drug–target information from the Drug–Gene Interaction database (DGIdb) [18], the Therapeutic Target Database (TTD) [19], and DrugBank [20]. The results showed that these epistatic gene pairs showed extensive connections to known pharmacological targets. Specifically, DGIdb-based analysis showed that 1040 epistatic gene pairs were mapped to genes with 572,426 drug–gene interaction records, including 121,508 records involving FDA-approved drugs and 14,212 records involving antineoplastic agents. The TTD-based analysis indicated that 398 epistatic genes were linked to 203,659 drugs, of which 739 were FDA-approved drugs. Similarly, DrugBank-based analysis identified 317 epistatic genes associated with 12,326 drugs. Further, by integrating FDA-approved combinatorial drug annotations from the DrugCombDB database [21], we identified 12 reported anticancer drug combinations whose target genes also appear in our epistatic analysis (Table 3). For example, the epistasis genes *TERT-FLT1* correspond to the doxorubicin-sorafenib drug combination against cancer [52].

Subsequently, we constructed and visualized a gene interaction network by integrating evidence from nominal-significant statistical epistatic pairs, gene co-expression, and documented protein–protein interactions. The analysis focused on highly connected hubs (degree > 3) and their immediate neighbors. This network encompassed multiple cancer-relevant genes, such as immune-related *HLA* family genes, *TAP2*, and *DEF8* (Figure 3E), suggesting that such functionally pivotal genes frequently emerge as central regulators within genetic interaction networks.

### 3.5. Survival Analysis Reveals Epistatic-Specific Prognostic Associations

To assess the clinical utility of epiSNPs and epiSNP pairs, we performed survival analysis based on overall survival (OS) data from UKBB using additive, recessive, and dominant models. A total of 57,750 epiSNP pairs showed nominal associations with patient overall survival (log-rank *p* < 0.05). After FDR correction, 7068 epiSNP pairs remained significantly associated with overall survival at FDR < 0.05.

To further evaluate whether these survival-associated epiSNP pairs provided prognostic information beyond individual SNP effects, we performed multivariable Cox proportional hazards regression. After excluding pairs for which the median survival time could not be estimated in any genotype group, 882 epiSNP pairs across the 16 cancer types were included in Cox regression models. Each model included the two SNP main effects, their interaction term, smoking status, and age. Sex was additionally included for cancers affecting both males and females, but not for sex-specific cancers. A total of 123 epiSNP pairs showed significant SNP–SNP interaction effects associated with OS. For example, in liver cancer, the epiSNP pair rs118179360 in *CTNNA3* and rs113349525 (located near *GOLGA6L1* and *TUBGCP5*) showed a significant interaction effect on OS (interaction *p* = 3.54 × 10^−4^, HR = 2.21, 95% CI: 1.43–3.42), whereas neither SNP showed a significant main effect (SNP1 *p* = 0.23; SNP2 *p* = 0.43) (Figure 3D, Supplementary Table S1). Notably, the interaction HR (2.21) was substantially larger than the main-effect HRs of either individual SNP (0.85 and 0.91), suggesting that the combined effect of these two variants confers a higher risk than either variant alone. Consistent with this finding, *CTNNA3* functions as a tumor suppressor in hepatocellular carcinoma by inhibiting Akt signaling, and its low expression is independently associated with poor survival [53].

### 3.6. Construction and Application of EpiSNPdb Database

**Figure 4:**
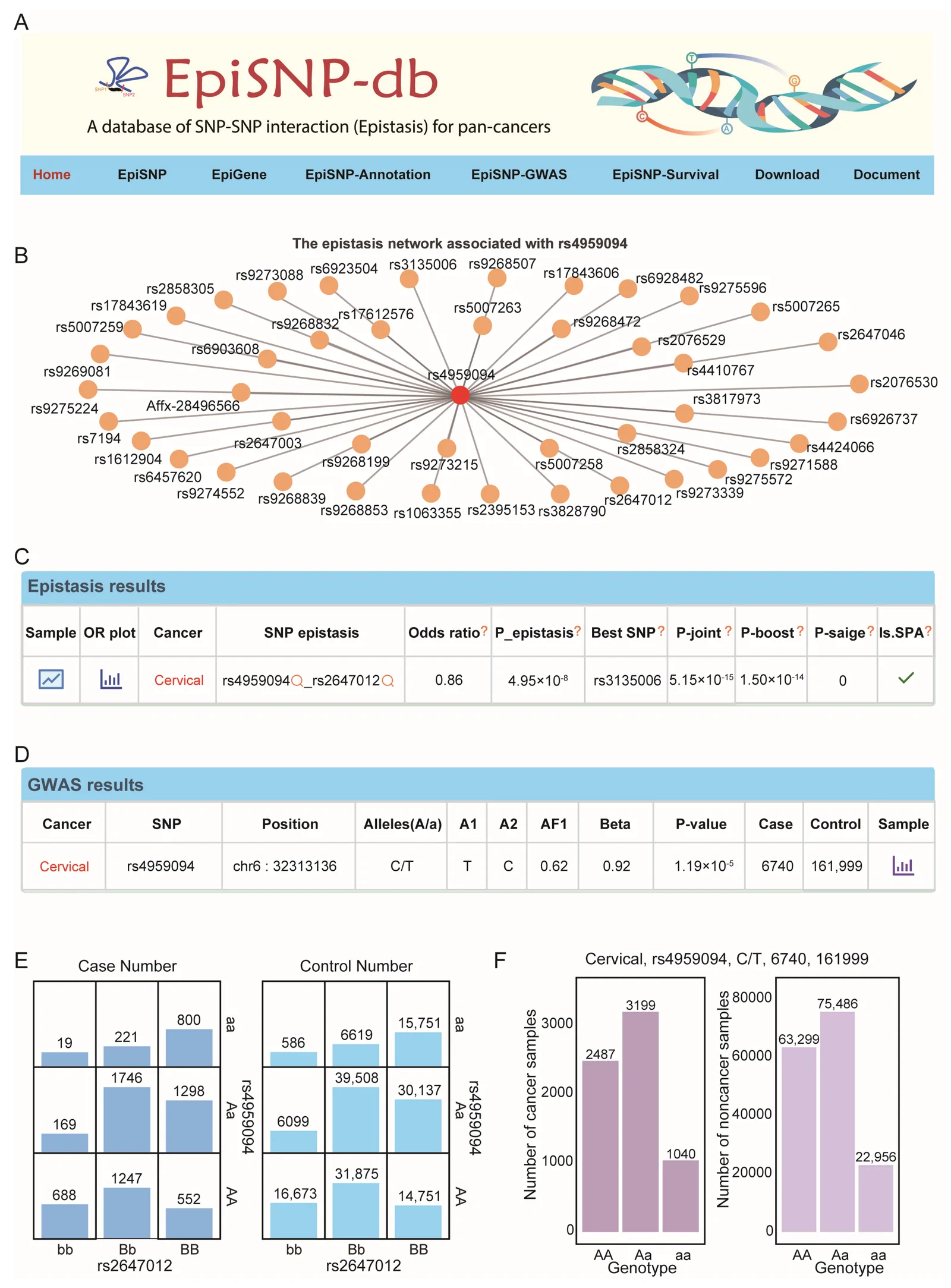
The interface of the EpiSNPdb database. (**A**) Overview of EpiSNPdb and its modules. (**B**) Epistasis network associated with rs12924101. (**C**) Output table shows epistatic pairs found by querying SNP rs4959094 in both the ‘Home’ and ‘EpiSNP’ modules. The question mark icons provide pop-up explanatory notes that describe the definition and biological meaning of each table column when hovering the mouse cursor. The search icon enables direct access to the corresponding SNP entry page in the dbSNP database upon clicking. (**D**) GWAS statistical output for rs4959094 in the ‘Home’ and ‘EpiSNP-GWAS’ modules. (**E**) The contingency table displays case and control sample distribution for epiSNP pair rs2647012_rs4959094 in the ‘EpiSNP’ module. (**F**) The bar graph displays case–control sample distribution across three SNP genotypes.

To facilitate the exploration of epistasis in cancer research, we developed EpiSNPdb (https://gonglab.hzau.edu.cn/EpiSNPdb/#/, accessed on 21 July 2026), a comprehensive database providing a comprehensive epistasis results across 16 cancer types. EpiSNPdb includes five core functional modules: EpiSNP, EpiGene, EpiSNP-Annotation, EpiSNP-GWAS, and EpiSNP-Survival (Figure 4A). Additionally, we provided a quick query on the ‘Home’ page for convenient accessibility of epistatic information across 16 cancer types. By entering a SNP of interest, a chromosomal region, an epiSNP pair, or a cancer type, users can obtain several results, including all epistatic interactions of the queried epiSNP (Figure 4B) as well as corresponding information of epiSNPs and epiSNP pairs (Figure 4C,D). For each epistatic interaction pair, EpiSNPdb provides the original GWAS *p*-values of the constituent SNPs, the epistasis test *p*-value, the SAIGE *p*-value, and the Benjamini–Hochberg FDR where applicable. Users can filter results on the web interface by setting custom thresholds for these metrics, allowing them to adjust the stringency of the candidate set according to their needs.

The ‘EpiSNP’ module provides detailed statistical data of epiSNP pairs, including OR values and *p*-values from three PLINK methods and SAIGE (Figure 4C). Bar charts show sample distribution and OR trend across nine genotype combinations (Figure 2E and Figure 4D), enabling direct assessment of epistatic effect in specific cancers. The ‘EpiGene’ module provides gene expression correlation analysis results of epistatic genes, presenting Spearman correlation coefficients (R value) and statistical significance (*p*-value < 0.05) and PPI confidence scores from the STRING database. Scatter plots of correlation analysis (Figure 3C) facilitate assessment of transcriptional coordination between epistatic genes. The ‘EpiSNP-Annotation’ module supports queries for individual epiSNPs or epiSNP pairs and outputs annotation information from ANNOVAR and PPI confidence scores. The ‘EpiSNP-GWAS’ module provides epiSNP GWAS results, including effect allele (A1), non-effect allele (A2), effect allele frequency (AF1), effect sizes (beta), and *p*-value. A bar graph visualizes the distribution of case and control sample sizes across three SNP genotypes (Figure 4F). The ‘EpiSNP-Survival’ module provides survival analysis results of individual epiSNPs and epiSNP pairs under three genetic models (dominant, recessive, and additive). KM survival curve visualizes the differences in survival duration across multiple genotypes.

All the data in the EpiSNPdb database are available on the ‘Download’ module. Moreover, EpiSNPdb provides detailed helpful documentation, including data collection methods, analysis tools, data sources, and contact information, enabling users to accurately understand and appropriately apply data.

## 4. Discussion

In this study, we developed EpiSNPdb, a comprehensive resource of genetic epistasis across 16 cancer types, based on large-scale UK Biobank data. Compared with previous epistasis research, our study involves larger case–control sample sizes and employs a more robust analysis procedure [11,14,39], enabling the identification of a substantial number of candidate epistatic interactions associated with cancer susceptibility and prognosis. As a resource-oriented database designed for discovery, EpiSNPdb prioritizes sensitivity to capture suggestive signals that may warrant further functional investigation. Among the 202,032 candidate epiSNP pairs, only 7762 were detected by all three PLINK methods. Specifically, logistic regression tests interaction with main effects, Boost regression screens for frequency deviations, and the joint-effects test compares genotype combinations. Because each method captures different aspects of epistasis, the low overlap reflects their complementarity.

To clarify the novelty and significance of EpiSNPdb, we compared its features with those of existing epistasis databases and interaction resources (Table 4).

To help users prioritize candidates, EpiSNPdb integrates orthogonal biological evidence from independent data sources, including gene co-expression in TCGA, protein–protein interactions in STRING, and published literature. As a discovery-oriented resource, the reported interactions are intended to serve as candidates for further investigation and independent validation rather than as confirmed biological associations.

Our study reveals the capacity of epistasis analysis to uncover novel genetic and prognostic insights beyond conventional single-variant approaches. From a susceptibility perspective, we identified 5929 epistatic SNP pairs whose individual SNPs were not significant in a genome-wide association study, thereby expanding the catalog of cancer-related susceptibility signals. From a prognostic perspective, 123 epiSNP pairs showed significant SNP–SNP interaction effects associated with OS in multivariable Cox regression. For example, an epistatic pair in *CTNNA3* was associated with overall survival in liver cancer. *CTNNA3* encodes alpha-3 catenin, a tumor suppressor that has been shown to inhibit Akt signaling in hepatocellular carcinoma. Experimental studies have shown that *CTNNA3* knockdown promotes proliferation and invasion of liver cancer cells, while its low expression is independently associated with poor survival [53]. Notably, the interaction effect conferred a higher risk than either individual SNP (interaction HR = 2.21, 95% CI: 1.42–3.42), whereas neither SNP showed a significant main effect. This suggests that the combined genotype of these two variants may disrupt CTNNA3-mediated tumor suppression more profoundly than either variant alone, providing a plausible mechanistic explanation for the observed epistatic effect on overall survival. These results suggest that interaction-level genetic signals may provide prognostic information beyond single-SNP effects. Given known links between germline variation and aggressive cancer phenotypes [54], our survival-related epiSNP pairs may serve as exploratory prognostic candidates.

Notably, the interaction effect conferred a higher risk than either individual SNP, indicating that epistatic interactions may capture risk combinations not evident from single-locus effects alone. These results suggest that interaction-level genetic signals may provide prognostic information beyond single-SNP effects. Given known links between germline variation and aggressive cancer phenotypes [54], our survival-related epiSNP pairs may serve as exploratory prognostic candidates.

Notably, most epiSNPs lie in intergenic and intronic regions, consistent with evidence that cancer susceptibility loci often act through gene-regulatory mechanisms, such as chromatin interactions and long noncoding RNAs, rather than direct coding effects [55,56]. This noncoding enrichment supports the biological relevance of these signals and motivates the functional annotation analyses described below.

Functional annotation analyses provide insights into potential biological contexts underlying epistasis. At the transcriptional level, 7152 gene pairs involved in epistatic interactions showed significant co-expression patterns. At the protein level, 12,326 epistatic gene pairs had documented PPI information, of which 2866 pairs exhibited high confidence scores [17]. These findings suggest that genetic epistatic signals may be associated with transcriptional coordination and protein interaction patterns. A representative example is the interaction between SNPs rs7788807 and rs9972533, which together strongly reduce lung cancer risk. Their corresponding genes, *FOXK1* and *SIN3A*, are co-expressed in tumors, and their proteins are known to physically interact. This observation aligns with established findings that the FOXK1–SIN3A complex functions as a transcriptional repressor that epigenetically silences oncogenic drivers and inhibits tumor-promoting signaling in lung cancer [49,50,51]. Specifically, *FOXK1* recruits *SIN3A* to the promoters of target genes such as *HIF1β* and *EZH2*, leading to histone deacetylation and transcriptional repression. The observed protective effect of the rs7788807–rs9972533 interaction is therefore consistent with a model in which the joint genotype enhances the tumor-suppressive activity of the FOXK1–SIN3A complex, providing a plausible mechanistic basis for this epistatic signal.

We found that hub genes in the epistatic network, such as *HLA* family genes, *TAP2*, and *DEF8*, were significantly enriched for genes with established functions in cancer. Specifically, the downregulation of HLA family genes in cancers has been reported to impair antigen presentation, which may contribute to tumor immune evasion through reduced T-cell recognition [57,58]. Similarly, *TAP2* serves as a critical node linking intrinsic oncogenic signaling with antitumor immunity. In most cancer types, downregulation of *TAP2* impairs antigen presentation and may contribute to immune evasion. Conversely, in breast cancer, elevated *TAP2* expression is associated with greater tumor aggressiveness [59,60,61]. Furthermore, *DEF8* primarily exerts tumor-suppressive effects through mechanisms including genetic association, negative regulation of autophagy, and modulation of lysosomal function [62,63]. These findings indicate that hub genes derived from epistatic networks are likely to exert important roles in cancer development, offering a systems-level strategy for prioritizing candidate drivers for further functional validation.

Some epistatic interactions exhibited a pattern in which alleles individually associated with reduced cancer risk, when combined, were associated with elevated risk. This reflects the non-additive nature of epistasis. The joint genotype can produce effects that differ in direction from the sum of individual effects. Such patterns may arise when two variants affect different steps of the same signaling pathway or act on pathways that oppose each other, and the combination disrupts the normal balance. However, these interpretations remain unconfirmed, and experimental studies are needed to determine the underlying mechanisms. Accordingly, candidate interactions should be interpreted together with independent lines of biological evidence, including gene co-expression, protein–protein interaction data, and published literature, rather than on the basis of statistical significance alone.

The identified epiSNP pairs may also offer exploratory leads for pharmacologic investigation, though their therapeutic relevance requires further validation. DGIdb-based analysis showed that 1040 epistatic gene pairs were mapped to genes with 572,426 drug–gene interaction records, including 121,508 records involving FDA-approved drugs and 14,212 records involving antineoplastic agents. Notably, our identified 12 epistatic pairs overlap with previously reported anticancer drug combinations. For example, the *TERT-FLT1* epistatic pair is targeted by the doxorubicin–sorafenib combination against cancer [52,64]. These findings provide potential connections between epistatic gene pairs and known drug targets. However, these computational annotations offer exploratory leads rather than direct evidence of therapeutic relevance, and further experimental validation is required.

Several limitations should be acknowledged. First, this study is restricted to germline genetic variants. Somatic mutations, which also contribute to cancer etiology, were not analyzed. Second, this study was restricted to individuals of White British ancestry, which may limit the generalizability of the findings. Third, cancer phenotypes in large-scale biobank data may be heterogeneous due to differences in diagnosis, classification, and clinical characteristics. Third, as a discovery-oriented resource, our results provide suggestive associations rather than definitive conclusions. We recommend users prioritize candidates with multi-dimensional evidence (e.g., high-confidence PPI) for further validation. Additionally, due to substantial missing data for tumor stage, treatment information, and comorbidities, we were unable to adjust for these clinical factors in our multivariable Cox regression models for overall survival. Consequently, the reported prognostic associations should be considered exploratory and may be influenced by unmeasured confounding. Moreover, the SAIGE filter was intentionally applied as a conservative screen. Consequently, the 71,649 SAIGE-significant interactions represent a high-confidence subset, and some genuine interactions with more complex patterns may not be included. Finally, the biological interpretation was mainly based on annotation-based evidence, which provides functional context but does not establish causal mechanisms. Experimental studies are needed to further clarify the biological relevance of these interactions.

## 5. Conclusions

In this study, we performed a systematic epistasis analysis across 16 cancer types and constructed EpiSNPdb, a user-friendly database that integrates statistical epistasis, genomic annotation, expression correlation, protein–protein interactions, and survival analysis. EpiSNPdb provides a valuable resource for the cancer genetics community and offers unique insights into the functional and potential clinical relevance of epistatic interactions. Its open accessibility is expected to accelerate epistasis research, refine cancer risk stratification, and ultimately support the design of personalized treatment approaches.

## Figures and Tables

**Table 1:** Sample size, number of epiSNP pairs and survival-related epiSNP pairs (nominal log-rank *p* < 0.05) in EpiSNPdb.

UKBB Cancer Type	Number of Cases	Number of Cancer-Free Samples	Number of epiSNP Pairs	Number of Survival-Related epiSNP Pairs
Breast	19,207	161,999	11,946	3150
Prostate	14,466	136,966	13,734	3853
Melanoma	8416	298,965	11,311	3135
Colon	6834	298,965	10,224	2768
Cervical	6740	161,999	30,174	7308
Lung	4960	298,965	20,715	3816
Bladder	4128	298,965	10,093	2881
Rectal	3142	298,965	10,253	3245
Uterine	2768	161,999	10,470	3103
Kidney	2314	298,965	10,530	3322
Ovarian	2182	161,999	9865	3464
Pancreatic	1668	298,965	10,336	3470
Esophagus	1609	298,965	10,531	3238
Stomach	1305	298,965	9631	2780
Brain	1157	298,965	11,339	4014
Liver	1122	298,965	10,880	4203

**Table 2:** FDR-significant epistatic interactions supported by multiple complementary lines of biological evidence.

Cancer Type	Gene Pair	epiSNP Pair	FDR	Co-Expression	STRING Score	Supporting PMID
Cervical	*HLA-E_HLA-B*	rs1264457_rs1131500	4.1 × 10^−2^	0.81	994	35323192; 32560316
Breast	*SETD9_MIER3*	rs2257505_rs189695	1.1 × 10^−3^	0.44	617	22993404; 19519208; 18437204; 19088016
Cervical	*HLA-DRA_HLA-DQA2*	rs3129876_rs4394270; rs3129876_rs4394270; rs3129878_rs4394270; rs3129881_rs4394270; rs3129882_rs9276427	1.3 × 10^−2^; 1.3 × 10^−2^; 2.3 × 10^−3^; 1.0 × 10^−2^; 5.0 × 10^−2^	0.65	879	31602270
Cervical	*HLA-DRA_HLA-DQB1*	rs3129882_rs1130375; rs3129882_rs35915063	6.0 × 10^−3^; 2.2 × 10^−3^	0.83	976	35342352; 34768100; 31602270
Cervical	*HLA-DRA_HLA-DQB2*	rs3129882_rs34988824; rs3129888_rs34988824; rs3129888_rs34988824	1.5 × 10^−3^; 2.5 × 10^−2^; 2.5 × 10^−2^	0.76	962	35153292; 34805135; 34923982; 34768100; 35342352

**Table 3:** Previously reported anticancer drug combinations corresponding to epistatic genes.

Gene 1	Drug 1	Gene 2	Drug 2	Source (PubMed ID)
*TERT*	doxorubicin	*FLT1*	sorafenib	30960820
*TERT*	doxorubicin	*FLT1*	sorafenib	24905399
*GPX5*	gemcitabine	*ALDH1A1*	cisplatin	27765756
*HLA-C*	gemcitabine	*EHMT2*	cisplatin	27765756
*PRRC2A*	gemcitabine	*EHMT2*	cisplatin	27765756
*MSH5*	gemcitabine	*EHMT2*	cisplatin	27765756
*ARHGEF10*	paclitaxel	*DOCK8*	carboplatin	26401644
*ALK*	carboplatin	*ATM*	olaparib	29750868
*ALK*	ceritinib	*ATM*	paclitaxel	28991240
*GATA3*	vincristine	*PRKD1*	quercetin	28463792
*GATA3*	vincristine	*PRKD1*	quercetin	21371568
*ESR1*	cisplatin	*WWOX*	gemcitabine	29614265
*ESR1*	olaparib	*MET*	palbociclib	30898650
*TET2*	olaparib	*CDKN2A*	palbociclib	30898650
*ALK*	pembrolizumab	*ATM*	ipilimumab	30885353
*KRAS*	sorafenib	*NF1*	vemurafenib	30844744

**Table 4:** Comparison of EpiSNPdb with existing epistasis databases and interaction resources.

Resource	Phenotype	Sample Size	Key Features	Data Freely Available
EpiSNPdb	16 cancer types	380,983	Cancer germline SNP–SNP epistasis integrated with survival analysis, FDA drug–target annotation, and an interactive web interface with custom filtering	https://gonglab.hzau.edu.cn/EpiSNPdb/, accessed on 28 June 2026
Epistasis Disease Atlas	8 complex diseases	22,469	Higher-order SNP epistasis (3-SNP, 4-SNP); focused on non-cancer diseases without survival or drug annotation	https://epistasis-disease-atlas.com/, accessed on 28 June 2026
SynLethDB v2.0	Pan-cancer and other diseases	Curated from literature, CRISPR screens, shRNA experiments	Gene-level synthetic lethal interactions queryable by cancer type or drug, with drug sensitivity data from curated experiments	https://synlethdb.sist.shanghaitech.edu.cn/, accessed on 28 June 2026
STRING v12	General (all organisms)	>14,000 organisms	Comprehensive protein–protein interaction network with confidence scores; not designed for SNP-level epistasis	https://string-db.org/, accessed on 28 June 2026

## Data Availability

EpiSNP-db is freely available to the public without registration or login requirements at https://gonglab.hzau.edu.cn/EpiSNPdb/#/ (accessed on 21 July 2026). The source code for data processing and database construction of EpiSNP-db is publicly available on GitHub at https://github.com/wuxh-create/EpiSNPdb (accessed on 21 July 2026). All publicly available datasets analyzed in this study were downloaded from their official repositories with corresponding versions and access dates specified below: STRING database: https://cn.string-db.org/ (accessed on 13 June 2025). Drug-Gene Interaction Database (DGIdb v5.0): https://dgidb.org (accessed on 20 October 2025). Therapeutic Target Database (TTD): https://ttd.idrblab.cn/ (accessed on 9 October 2025). DrugBank (v6.0): https://go.drugbank.com (accessed on 10 October 2025). DrugCombDB: http://drugcombdb.denglab.org/main (accessed on 10 October 2025). UK Biobank (UKB): The UK Biobank dataset utilized in this study is not publicly open-access. It is only available to qualified researchers through formal project application and official data access approval via the UK Bi-obank portal (https://www.ukbiobank.ac.uk/).

## Supplementary Materials

The following supporting information can be downloaded at: https://www.mdpi.com/article/10.3390/cimb48080753/s1.

## Abbreviations

The following abbreviations are used in this manuscript:

epiSNP	Epistatic single nucleotide polymorphism
PPI	Protein–protein interaction
GWAS	Genome-wide association study
UKBB	UK Biobank
DGIdb	Drug–Gene Interaction database
TTD	Therapeutic Target Database
OS	Overall survival
MAF	Minor allele frequency
OR	Odds ratio
